# Decreased frontal gyrification correlates with altered connectivity in children with autism

**DOI:** 10.3389/fnhum.2013.00750

**Published:** 2013-11-08

**Authors:** Marie Schaer, Marie-Christine Ottet, Elisa Scariati, Daniel Dukes, Martina Franchini, Stephan Eliez, Bronwyn Glaser

**Affiliations:** ^1^Stanford Cognitive and Systems Neuroscience Laboratory, Stanford University School of Medicine, Palo AltoCA, USA; ^2^Office Médico-Pédagogique, Department of Psychiatry, University of Geneva School of MedicineGeneva, Switzerland; ^3^Cognitive Science Centre, University of NeuchâtelNeuchâtel, Switzerland; ^4^Swiss Center for Affective Sciences, University of Geneva Geneva, Switzerland; ^5^Department of Genetic Medicine and Development, University of Geneva School of MedicineGeneva, Switzerland

**Keywords:** cortical folding, cerebral morphometry, tractography, neuroimaging, autism spectrum disorder

## Abstract

The structural correlates of functional dysconnectivity in autism spectrum disorders (ASD) have been seldom explored, despite the fact that altered functional connectivity is one of the most frequent neuropathological observations in the disorder. We analyzed cerebral morphometry and structural connectivity using multi-modal imaging for 11 children/adolescents with ASD and 11 matched controls. We estimated regional cortical and white matter volumes, as well as vertex-wise measures of cortical thickness and local Gyrification Index (*l*GI). Diffusion Tensor Images (DTI) were used to measure Fractional Anisotropy (FA) and tractography estimates of short- and long-range connectivity. We observed four clusters of *l*GI reduction in patients with ASD, three were located in the right inferior frontal region extending to the inferior parietal lobe, and one was in the right medial parieto-occipital region. Reduced volume was found in the anterior corpus callosum, along with fewer inter-hemispheric frontal streamlines. Despite the spatial correspondence of decreased gyrification and reduced long connectivity, we did not observe any significant relationship between the two. However, a positive correlation between *l*GI and local connectivity was present in all four clusters in patients with ASD. Reduced gyrification in the inferior fronto-parietal and posterior medial cortical regions lends support for early-disrupted cortical growth in both the mirror neuron system and midline structures responsible for social cognition. Early impaired neurodevelopment in these regions may represent an initial substrate for altered maturation in the cerebral networks that support complex social skills. We also demonstrate that gyrification changes are related to connectivity. This supports the idea that an imbalance between short- and long-range white matter tracts not only impairs the integration of information from multiple neural systems, but also alters the shape of the brain early on in autism.

## Introduction

Autism is a heterogeneous disorder characterized by a triad of symptoms including impairments in social interactions, delayed development of spoken language, and repetitive patterns of behavior. To satisfactorily account for the observed clinical heterogeneity in autism, the name “autism spectrum disorder” (ASD) is commonly used to convey the associated clinical manifestations that vary in severity along a continuum of autistic traits. Most epidemiological records estimate the global prevalence of ASD at 1 in 160 individuals (Elsabbagh et al., 2012), with some studies reporting rates as high as 1 in 88 children (Centers for Disease Control and Prevention, 2008). Understanding the neurobiological bases of this pervasive developmental disorder, which highly impacts the societal integration and professional achievements of affected persons, is one of the main motivations driving the prolific research on the disorder.

Structural and functional neuroimaging studies on ASD are particularly abundant. Early morphometric studies reported increased brain volume, but decreased total volume thereafter, during the first years of life in patients with ASD compared to healthy controls (Courchesne et al., 2001; Courchesne, 2004). This pattern of early overgrowth followed by degenerative change has been explained by a failure to refine the cerebral circuitry through the adaptive, experience-driven processes normally occurring during childhood (Courchesne et al., 2011). Indeed, there is a large amount of evidence for disrupted organization of cerebral networks in children, adolescents and adults with autism. Structural brain imaging studies reported increased white matter volume in regions corresponding to local cortico-cortical connections, and only minor changes, or even sometimes decreased volumes, in regions corresponding to long-range or inter-hemispheric connections (reviewed in Minshew and Williams, 2007). Increased local connectivity and reduced distant connectivity was further corroborated by post-mortem examinations (Zikopoulos and Barbas, 2010) and by functional studies using EEG (Barttfeld et al., 2011). Finally, several fMRI studies measuring functional connectivity using resting-state paradigms observed decreased long-range functional connectivity in children or young adults with ASD (Kennedy and Courchesne, 2008; Assaf et al., 2010; Weng et al., 2010; von dem Hagen et al., 2013). Despite the plethora of evidence for disrupted structural and functional connectivity and a growing body of research demonstrating morphometric differences in the brains of patients with autism; there are surprisingly few integrated studies that show how differences in cerebral morphology and connectivity fit together. Adding to our knowledge of the relationships between different anatomical impairments should elucidate the mechanisms underlying brain alterations in autism.

It is accepted that cortical folding reflects a person’s prenatal development (Regis et al., 2005) (as well as events from the first months of post-natal life (Schaer et al., 2009; Haukvik et al., 2011)). It follows that measuring the shape of the cortex, using three-dimensional cortical reconstructions, provides us with insight into early brain development. Although the processes underlying the creation of specific sulcal patterns are poorly understood, existing theories point to the determinants of early cortical folding. Initial hypotheses proposed that gyrification results from mechanical forces intrinsic to the cortex (Richman et al., 1975; Welker et al., 1990). However, more recent theories consider cortical shape as a product of underlying patterns of connectivity, implicating alterations to both connectivity and cortical folding, both of which are highly relevant in autism. The tension-based model of convolutional development (Van Essen, 1997) postulates that strongly interconnected cortical regions are pulled towards one another during embryological development, resulting in both compact and streamlined wiring of the nervous system. According to this model, disturbed gyrification in the adult human brain reflects abnormal patterns of white matter connectivity. Measuring gyrification abnormalities at any age may signal early adverse events and contribute to our understanding of both the timing and the nature of brain alterations in neurodevelopmental disorders.

Previous studies have noted alterations to cortical shape in autism (Levitt et al., 2003; Nordahl et al., 2007; Shokouhi et al., 2012), and some have used the Gyrification Index (GI; Hardan et al., 2004; Casanova et al., 2009; Kates et al., 2009; Jou et al., 2010; Meguid et al., 2010). Given that the cortex grows primarily through radial expansion (Rakic, 1988), the GI was specifically designed to identify early defects in cortical development. However, all but one (Wallace et al., 2013) existing studies quantifying GI in patients with ASD used two-dimensional or global approaches, which do not allow for the identification of focal differences. By contrast, the local Gyrification Index (*l*GI; Schaer et al., 2008) has been shown to provide reliable estimates of GI with fine-grained resolution in many conditions (Zhang et al., 2009; Juranek and Salman, 2010; Zhang et al., 2010; Palaniyappan and Liddle, 2012; Palaniyappan et al., 2011; Ronan et al., 2011; Thesen et al., 2011; Srivastava et al., 2012).

In the present study, we sought to combine advanced multi-modal techniques in a small individually-matched group of ASD and healthy controls in order to simultaneously examine alterations in gyrification and structural connectivity. We first propose an exploratory analysis of the morphometry of gray and white matter structure, and of white matter connectivity. For that purpose, we use the T1-weighted imaging to quantify the total cerebral and cerebellar gray and white matter volumes, the regional cortical and white matter volumes, and to measure cortical thickness and *l*GI at thousands of points across the hemisphere. We also exploit Diffusion Tensor Imaging (DTI) to quantify voxel-wise alterations in white matter microstructure and use tractography to provide estimates of structural connectivity. In subsequent analyses, we aim at integrating the morphometric and the connectivity findings. In line with the Van Essen’s tension-based morphogenesis hypothesis, we expect to observe that regions with altered gyrification in autism corresponds to areas with aberrant patterns of short- and long-range connectivity, as quantified using tractography.

## Materials and Methods

### Participants

#### Patients with ASD

Eleven children and adolescents with ASD participated in the current study (8 males). The group had an average age of 12.9 ± 2.7 years (range 9.3–17.4) and an average full-scale IQ (using the Wechsler WISC-III (Wechsler, 1991)) score of 79.4 ± 18.1 (range 51–105). Participants were recruited with the help of local associations, therapeutic schools, and a local ASD diagnostic clinic. Once participants contacted us to express interest in the study, a detailed medical history, including details about their diagnosis were taken. Individuals with known genetic disorders, as well as malformations and birth defects, were excluded. An initial appointment was then set to reconfirm participants’ diagnoses using the autism diagnostic interview-revised (ADI-R) interview with one or both parents. The group of patients with ASD had the following scores on the ADI-R (Le Couteur et al., 2007): social interaction: 15.7 ± 7.9, communication: 12.4 ± 6.6, restricted and repetitive behaviors: 5.7 ± 3.3. The ADI-R was followed by an autism diagnostic observation scale (ADOS; Lord et al., 2009), which was conducted by a research-reliable clinician from the institution’s ASD diagnostic clinic. The parents of all participants also filled in the Social Communication Questionnaire (SCQ; Berument et al., 1999).

#### Control participants

The comparison group was comprised of 11 healthy controls, individually matched with each patient for gender and age. The control group had an average age of 12.7 ± 2.7 (range 8.7–16.8). There was no difference in mean age between patients and controls (*p* = 0.897). The average full-scale IQ of the control group was 110.5 ± 13.3 (range 88–129).

Written informed consent was received from all subjects and their parents in accordance with protocols approved by the local ethics committee.

### Image processing

Cerebral magnetic resonance images were acquired using a Siemens Trio 3T scanner at the Geneva Center for Biomedical Imaging (CIBM). A set of T1-weighted 3D volumetric images was acquired as a series of 192 contiguous coronal slices, with a voxel size of 0.86 × 0.86 × 1.1 mm (repetition time (TR) = 1200 ms, echo time (TE) = 3 ms, flip angle = 8°). DTI were acquired on the same day as a series of 64 axial slices with 30 directions, with a voxel size of 2 × 2 × 2 mm (b0 = 1000 ms, TR = 8300 ms, TE = 82 ms, flip angle = 90°).

#### Cortical reconstruction

The T1-weighted images were used to create cortical reconstruction and volumetric segmentation using the *FreeSurfer* package (Martinos Center for Biomedical Imaging, Massachusetts General Hospital, Boston^1^). Briefly, the processing was comprised of removing non-brain tissue, executing automatic segmentation of the subcortical gray matter structures, and extracting cortical surface (Dale et al., 1999; Fischl, 2012). Both intensity and continuity data from the entire three-dimensional MR volume are used in the segmentation procedures, thus producing accurate representations of cortical thickness and volumes. These procedures have been validated against histological studies (Rosas et al., 2002) and have been shown to be reliable across scanner models and field strengths (Han et al., 2006). At the end of the reconstruction process, the following volumes were available: total cerebral gray and white matter volumes, cerebellar gray and white matter volumes, corpus callosum volume, and the volumes of subcortical structures including thalamus, putamen, pallidum, caudate nucleus, as well as amygdala and hippocampus.

#### Regional cortical volumes

Subsequent to cortical reconstruction, the cortex was also subdivided into units based on gyral and sulcal structures (Desikan et al., 2006). This parcellation method has been shown to be both valid and reliable, with high intra-class correlation coefficients between the manual and automated procedures for both cortical volume estimates and region boundaries. The parcellation produces 34 cortical regions subdivided into 11 frontal regions, 9 temporal regions, 5 parietal regions, 4 occipital regions, 4 parts of the cingulate cortex, and one label for the insula.

#### Regional white matter volumes

The parcellation of the cortical gray matter was subsequently used to subdivide the underlying white matter as described in Salat et al. (2009), a Voronoi diagram was created in the white matter voxels based on the distance to the nearest parcellation label, using a distance constraint of 5 mm. As a result of this process, regional white matter volumes were available for each of the 34 regions corresponding to the aforementioned gyral labeling.

The corpus callosum was also identified and subdivided into 5 portions along its anteroposterior axis, according to procedures detailed in Rosas et al. (2010). The volume of the corpus callosum was measured for each of the 5 portions (anterior, mid-anterior, center, mid-posterior and posterior) on a 5 mm lateral extent centered on the mid-sagittal place.

#### Cortical thickness and cortical gyrification

Cortical thickness was measured in the native space of the images, as the shortest distance between the white (gray-white boundary) and the pial (gray-CSF interface) surfaces. As a result, cortical thickness values with submillimeter accuracy were available at more than 150,000 different points over each hemisphere resolution (Fischl and Dale, 2000). Finally, based on the outer cortical surface reconstruction (pial surface), *l*GI was measured at thousands of points across each hemisphere using previously validated algorithms (Schaer et al., 2008). *l*GI is a surface-based measurement of the degree of cortical folding that iteratively quantifies the amount of cortex buried within the sulcal folds in the surrounding circular region.

Inter-subject comparison of the cortical thickness and gyrification values is achieved through spherical registration of the surfaces that minimizes metric distortion and allows for a highly reliable point-to-point comparison of cortical thickness between groups (Fischl et al., 1999).

#### Tract-based spatial statistics of the white matter structure

The DTI images were used for voxelwise statistical analysis of the Fractional Anisotropy (FA) using Tract-Based Spatial Statistics (TBSS; Smith et al., 2006), which is part of FSL software.^2^ First, FA images were created by fitting a tensor model to the raw diffusion data using algorithms embedded in the FDT toolbox, followed by skull stripping. As described in the original protocol (Smith et al., 2006, 2007), subjects’ FA data were then aligned into a common space using nonlinear registration. Next, the mean FA image was created and thinned to create a mean FA skeleton that represents the centers of all tracts common to the group. Each subject’s aligned FA data were then projected onto this skeleton and the resulting data were fed into voxelwise cross-subject statistics.

#### Tractography analyses

To relate the cortical anatomy with the underlying architecture of white matter fibers, we used tools embedded in the Connectome Mapping Toolkit (Daducci et al., 2012).^3^ Briefly, registration between the T1-weighted and DTI images was completed using the *bbregister* function of FreeSurfer. The DTI images were processed with Diffusion Toolkit software^4^ using the deterministic streamline algorithm (Mori et al., 1999) to obtain tractographic reconstruction of white matter bundles.

In the present study, we used the number of streamlines to quantify two different aspects of connectivity. First, we measured the amount of fibers connecting the homologous lobe between each hemisphere. The inter-hemispheric fibers obtained by this method represent a simple way to define long-range connectivity without having to define an arbitrary length threshold. To obtain the inter-hemispheric frontal fibers, we retained all streamlines connecting cortical regions comprised in the frontal lobe, as defined in the Desikan parcellation (Desikan et al., 2006). To select the streamlines corresponding to inter-hemispheric parietal streamlines, we repeated the process with cortical regions corresponding to the parietal lobe. Finally, streamlines connecting the temporal and occipital cortical regions were considered together for this analysis, given the small amount of inter-hemispheric fibers connecting these two lobes. As a result, three different variables summarizing one aspect of long-range connectivity were studied: the number of inter-hemispheric frontal, parietal, and temporo-occipital streamlines. Second, we measured the connectivity within each lobe (i.e., the number of streamlines connecting one lobe to itself) as an estimate of short-connectivity that also doesn’t require any arbitrary length threshold.

### Statistical analyses

#### Volumetric analyses

We used ANCOVA to compare cerebral, cerebellar and subcortical volumes between groups, including age and gender as covariates. To identify potential regional cortical alterations, we subsequently applied a MANCOVA on the 34 gyral regions in each hemisphere by entering diagnosis as the fixed factor, and both age and gender as covariates. Potential changes in regional white matter volumes were examined by doing a MANCOVA on the five corpus callosum regions, and another MANCOVA on the 34 regional white matter volume in each hemisphere. All the MANCOVA were performed with diagnosis as the fixed factor and both age and gender as covariates.

#### Cortical thickness and gyrification analyses

The comparisons of cortical thickness and gyrification over the whole brain used the *fsaverage* template included in the FreeSurfer distribution. Cortical thickness maps were smoothed using a full width at half maximum (FWHM) kernel of 10 mm. As *l*GI is already smooth (the degree of smoothness in our *l*GI data corresponds to a smoothing kernel of 10 mm), the data were not additionally smoothed prior to statistical analyses. Statistical analyses employed a General Linear Model (GLM) to estimate the effect of diagnosis, age and gender on thickness or gyrification at each cortical point. Cortical thickness or gyrification changes with age were fitted using a linear model. All results were corrected for multiple comparisons using the Monte Carlo simulation at the cluster level at the corrected significance threshold of *p* < 0.05.

#### Tract-Based Spatial Statistics of the white matter structure

TBSS voxel-wise analyses were carried out across subjects for each point of the common skeleton. As our population was comprised of children and adolescents, the mean FA volume provided by FMRIB software library (FSL) (“FMRIB58”) based on 58 adult brains was not optimal. Therefore, we chose the recommended alternative, using the “most typical” subject in our sample, to process the statistics. Local FA differences between patients and controls were tested for significance using a GLM. The skeleton-based approach has the advantage of reducing the number of statistical tests performed by reducing the number of voxels being compared. Nevertheless, we performed a permutation-based approach to control for “Family-Wise Error” (FWE; Nichols and Holmes, 2002). The options we used in the statistic TBSS pipeline were the most recommended ones: the Threshold-Free Cluster Enhancement (TFCE) option and a number of permutations at 500 (see the TBSS user guide on http://fsl.fmrib.ox.ac.uk/fsl/fslwiki/TBSS). A post-hoc *t-*test was ultimately conducted, comparing individual measurements of axial and radial diffusivity in clusters of significant between-group FA differences.

#### Tractography analyses

We used ANCOVA to compare the total number of streamlines with the total number of inter-hemispheric streamlines between patients with ASD and controls, while correcting for age and gender. We then conducted two MANCOVA to quantify potential differences between groups for the number of inter-hemispheric streamlines (long-range connectivity) and the number of streamlines connecting each lobe with itself (short-range connecti­vity), with age, gender and the total number of streamlines as covariates.

#### Correlations between gyrification and connectivity

We conducted partial correlations between the average *l*GI in each cluster of between-group differences and measures of short- and long-range connectivity, while correcting for the effects of age, gender and total number of fibers. For long-range connectivity, we correlated *l*GI for each cluster with the inter-hemispheric fibers corresponding to the lobe where the largest part of the cluster was located. For the short-range connectivity, we correlated *l*GI with the number of intra-lobar fibers in the lobe where the largest part of the cluster was located. These partial correlations were conducted separately in ASD and control groups. Given that these correlations were based on our *a priori* hypothesis postulating a relationship between gyrification and underlying connectivity, we did not correct for multiple comparisons.

#### Correlations with the clinical phenotype

Finally, we explored how the neuroanatomical differences observed between the two groups may be related to the clinical outcome. For that purpose, we conducted partial correlations between neuroanatomical variables and the scores obtained at the ADI-R and in the SCQ, correcting for age and gender. In addition, variables measuring the number of streamlines between regions of interest were also corrected for the total number of streamlines. Correlations with clinical phenotype were not corrected for multiple comparisons.

## Results

### Volumetric analyses

We did not observe any significant differences between the cerebral, cerebellar and subcortical volumes of the groups (all *p* > 0.386). Upon further examination of the 34 cortical parcel volumes, we did not detect any significant patterns of change (left: Wilks Lambda: 0.042, *p* = 0.612; right: Wilks Lambda: 0.028, *p* = 0.517), nor did we detect pattern differences in the 34 subcortical white matter regions (left: Wilks Lambda: 0.001, *p* = 0.087; right: Wilks Lambda: 0.037, *p* = 0.585). Despite the trend for a significant pattern of between-group differences in the MANCOVA corresponding to the left white matter subregions, none of the individual subregions revealed any significant difference in the post-hoc analysis.

**Figure 1:**
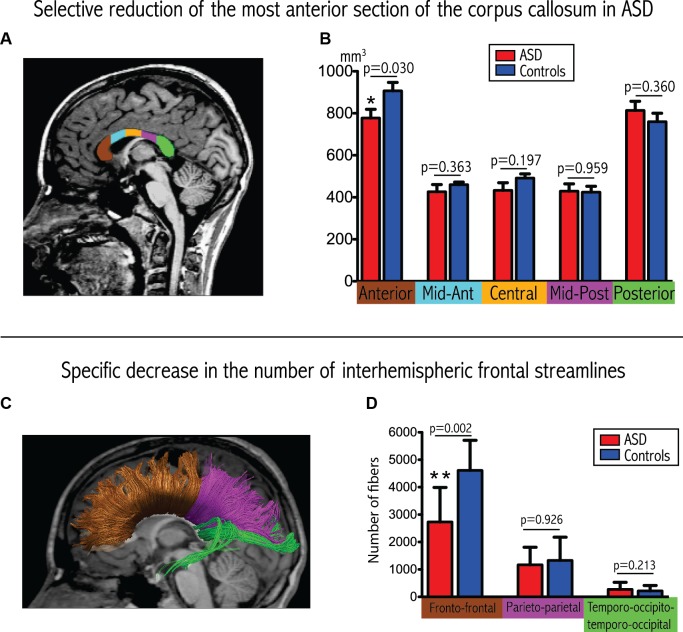
**Convergent evidence for disrupted connectivity at the level of inter-hemispheric frontal connectivity in ASD**. **(A)** Example of subdivision of the five sections of the corpus callosum displayed on a mid-sagittal slice. **(B)** Boxplots depicting volumetric measurements for the five sections in the two groups. The *p*-values are extracted from the MANCOVA (with correction for age and gender). **(C)** Example of tractographic reconstruction where the inter-hemispheric fibers are subdivided into three groups according to the cortical regions that they connect. **(D)** Boxplots comparing the number of streamlines connecting homologous lobes. The *p*-values are extracted from the MANCOVA (correction for age, gender and total number of streamlines).

Results from the corpus callosum analysis are depicted in Figures 1A, B. We observed significant group differences among the five sub-regions of the corpus callosum (Wilks Lambda: 0.354, *p* = 0.007, F = 5.11), with a selective reduction in the most anterior part of the corpus callosum in the ASD group compared to controls (*p* = 0.030). These results remained significant when covarying for total intracranial volume or total white matter volume instead of age and gender, as well as for total white matter volume, age and gender.

### Vertex-wise analyses

**Figure 2:**
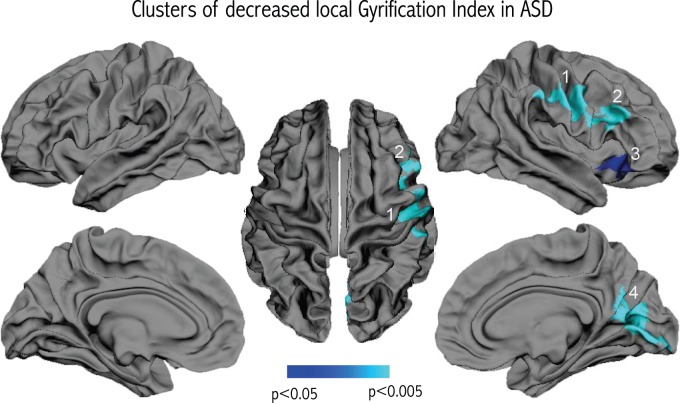
**Results of the vertex-wise comparison of *l*GI between groups**. Four clusters of reduced *l*GI were observed in the group of children and adolescents with ASD compared to controls after correcting for multiple comparisons.

We did not observe any significant differences in cortical thickness related to diagnosis. However, vertex-wise comparisons of gyrification revealed four clusters of significant *l*GI reduction in patients with ASD compared to controls that remained significant after correcting for multiple comparisons. As shown in Figure 2, the clusters were all located in the right hemisphere, in the inferior parietal region, the lower part of the precentral gyrus, the inferior frontal gyrus, and the medial parieto-occipital region (cuneus/precuneus).

### TBSS analyses

**Figure 3:**
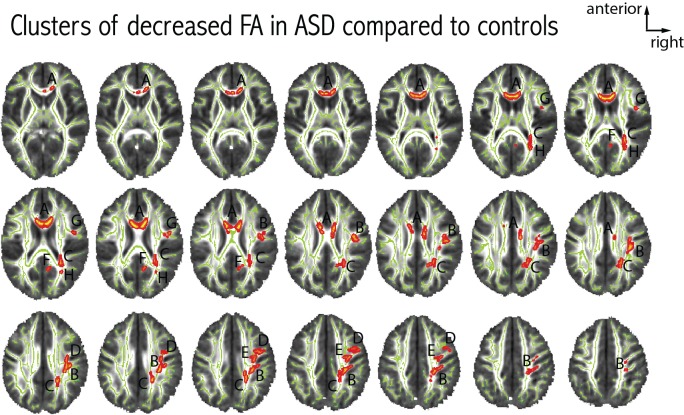
**Differences in FA between patients with ASD and controls**. Eight clusters of decreased FA in patients with ASD were observed at *p* < 0.05 (corrected for multiple comparisons). Each cluster was attributed a letter according to decreasing size. The clusters were centered at the following MNI coordinates: A: *x* = 81, *y* = 145, *z* = 90 (939 voxels); B: *x* = 52, *y* = 104, *z* = 104 (725 voxels); C: *x* = 66, *y* = 78, *z* = 101 (470 voxels); D: *x* = 57, *y* = 106, *z* = 129 (186 voxels); E: *x* = 46, *y* = 131, *z* = 112 (137 voxels); F: *x* = 82, *y* = 70, *z* = 93 (70 voxels); G: *x* = 43, *y* = 128, *z* = 90 (42 voxels); H: *x* = 60, *y* = 65, *z* = 88 (23 voxels). We did not observe any clusters with increased FA in patients with ASD compared to controls.

We found eight clusters of decreased FA in patients with ASD as compared to controls. The largest cluster of difference was located in the anterior part of the corpus callosum. The remaining seven clusters were located in the right hemisphere, no cluster of FA difference was seen in the left hemisphere. Figure 3 further details the distribution, location and size of these clusters. When comparing axial and radial diffusivity measurements in the clusters where FA significantly differed between patients with ASD and controls, we observed a significant between-group difference for axial diffusivity in only one cluster (cluster H, patients: 9.44e^-3^ ± 6.73e^-5^, controls: 8.96e^-3^ ± 4.59e^-5^, *p* = 0.03), whereas significantly decreased radial diffusivity was observed in all eight clusters in patients with ASD compared to controls (all *p* < 0.003).

### Tractographic analyses

We did not observe any difference in the total number of streamlines reconstructed from the DTI images (ASD: 103021 ± 10883, controls: 107331 ± 12769, *p* = 0.341).

We observed a significant reduction in the total number of inter-hemispheric fibers in patients with ASD (ASD: 4293 ± 1834, controls: 6276 ± 1771; *p* = 0.033, F = 5.405). Furthermore, and as demonstrated in Figures 1C, D, we also observed a significant between-group difference in the regional pattern of inter-hemispheric fibers (MANCOVA covarying out the effect of age, gender and total number of fibers: Wilks Lambda: 0.478, *p* = 0.010, *F* = 5.47), showing a selective reduction in the number of inter-hemispheric frontal fibers (*p* = 0.002), with a selective reduction in the number of inter-hemispheric frontal fibers (*p* = 0.002), but no significant differences in the inter-hemispheric parietal and temporo-occipital fibers.

No difference in the pattern of short-range connectivity was observed between the group of patients with ASD and controls (Wilks Lambda: 0.600, *p* = 0.594).

### Correlations between gyrification and connectivity

**Figure 4:**
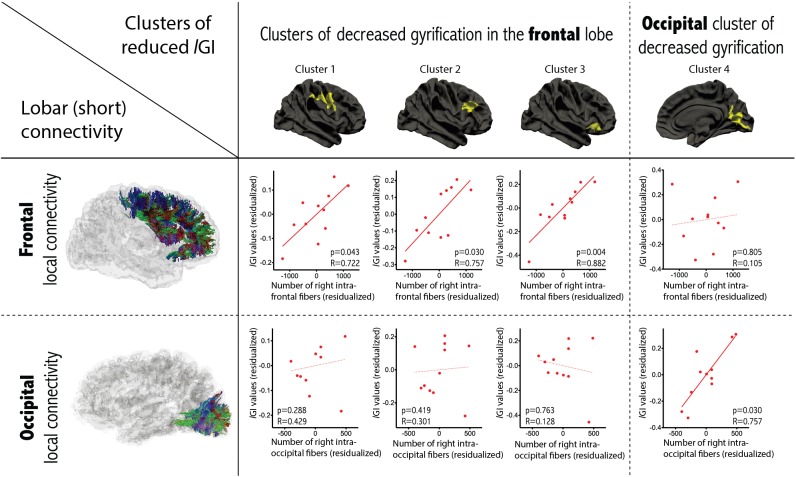
**Correlations between gyrification and short-range connectivity within the group of patients with ASD**. Partial correlations accounting for age, gender and total number of streamlines were conducted between *l*GI in the cluster of between-group differences and the number of intra-lobar streamlines in the lobe where most of the cluster was located. Significant positive correlations were observed only in the corresponding lobe (dashed lines report non-significant relationships).

Examining the relationship between gyrification and connectivity, we did not observe any significant relationships between the three clusters located in the frontal lobe and the number of inter-hemispheric frontal streamlines or in the occipital cluster and the number of inter-hemispheric occipital streamlines, in either diagnostic group. However, we found positive correlations between *l*GI and the variables measuring short-range connectivity, in the ASD group only. As depicted in Figure 4, we observed a significant positive correlation between *l*GI in all three right frontal clusters and the number of streamlines connecting the right frontal lobe to itself (*p* = 0.043, *p* = 0.030 and *p* = 0.004 for clusters 1, 2 and 3 respectively as numbered on Figure 2). We also observed a positive correlation between *l*GI in the right occipital cluster (cluster 4 on Figure 2) and the number of streamlines connecting the right occipital lobe to itself (*p* = 0.010, R = 0.836). The spatial correspondence of the positive correlation between gyrification and short-range connectivity is further supported by the absence of a significant correlation between the three frontal clusters of *l*GI differences and the occipital connectivity, and the absence of a significant correlation between the occipital *l*GI and the frontal connectivity (as depicted in Figure 4 with dashed lines).

### Correlations with the clinical phenotype within ASD

Results of the exploratory correlations between clinical scores at the ADI and SCQ and all variables that showed between-group differences are presented in Table 1. Briefly, *l*GI in the posterior cluster (number 4 in Figure 2) negatively correlated with the total score obtained on the ADI-R (R = −0.737, *p* = 0.024), and the reciprocal social interaction (R = −0.726, *p* = 0.027) and communication (R = −0.773, *p* = 0.015) domains from the ADI-R. Furthermore, the ADI-R scores in the domain of restrictive and stereotyped patterns of behaviors negative correlated negatively with the number of inter-hemispheric fibers (R = −0.784, *p* = 0.021) and with the number of inter-hemispheric frontal fibers (R = −0.779, *p* = 0.023). None of the neuroanatomical variables correlated with the total score obtained on the SCQ.

**Table 1:** **Correlations between clinical scores and neuroanatomical variables with between-group differences in participants with ASD**.

	**ADI: Total score**	**ADI: Reciprocal Social Interactions Score**	**ADI: Communication Score**	**ADI: Restrictive & stereotyped patterns of behavior**	**ADI: Early Anomalies in Development**	**SCQ: Total Score**
*Gyrification*						
*l*GI in cluster 1	−.005 (*p* = 0.989)	−.216 (*p* = 0.577)	.030 (*p* = 0.940)	.497 (*p* = 0.173)	.056 (*p* = 0.887)	.076 (*p* = 0.845)
*l*GI in cluster 2	−.136 (*p* = 0.728)	−.386 (*p* = 0.305)	−.020 (*p* = 0.959)	.451 (*p* = 0.223)	.041 (*p* = 0.917)	−.181 (*p* = 0.641)
*l*GI in cluster 3	−.037 (*p* = 0.925)	−.292 (*p* = 0.446)	.045 (*p* = 0.908)	.494 (*p* = 0.177)	.146 (*p* = 0.708)	−.117 (*p* = 0.764)
*l*GI in cluster 4	**−.737 (*p* = 0.024)**	**−.726 (*p* = 0.027)**	**−.773 (*p* = 0.015)**	−.043 (*p* = 0.912)	.205 (*p* = 0.597)	−.541 (*p* = 0.132)
*Volumetric measurements*						
Anterior section of the corpus callosum	.482 (*p* = 0.189)	.537 (*p* = 0.136)	.649 (*p* = 0.059)	−.325 (*p* = 0.394)	−.419 (*p* = 0.262)	.315 (*p* = 0.409)
*Tractographic measurements*						
Number of inter-hemispheric streamlines	−.345 (*p* = 0.402)	−.165 (*p* = 0.697)	−.317 (*p* = 0.445)	**−.784 (*p* = 0.021)**	.544 (*p* = 0.163)	−.682 (*p* = 0.063)
Number of inter-hemispheric frontal streamlines	−.390 (*p* = 0.339)	−.241 (*p* = 0.566)	−.309 (*p* = 0.457)	**−.779 (*p* = 0.023)**	.392 (*p* = 0.337)	−.681 (*p* = 0.063)

This table provides *R*-values from partial correlations. Significance level is given in parentheses. Partial correlations accounted for an effect of age and gender on gyrification and volumetric measurements, as well as for an additional effect of total number of streamlines on tractographic measurements. Significant correlations at *p* < 0.05 (uncorrected) are highlighted in bold.

## Discussion

In this study, we applied neuroimaging techniques using T1-weighted and DTI images in the interest of quantifying morphometric and connectivity differences in a group of children and adolescents with ASD. We observed: (a) decreased gyrification in the right inferior frontal region extending into the inferior parietal region and in the medial parieto-occipital region of patients with ASD as compared to controls, the latter of which was related to the severity of social communications deficits in the group of ASD; (b) convergent evidence from three different analyses for altered long-range connectivity at the level of inter-hemispheric frontal fibers: volumetric reduction of the anterior corpus callosum, reduced FA in the anterior corpus callosum, and a decreased number of virtual streamlines connecting homologous frontal lobes which further correlated with the severity of restrictive/repetitive behaviors; (c) further reduced FA in seven clusters of the right hemisphere of patients with ASD compared to controls; and (d) a positive correlation between *l*GI in the clusters of between-group differences and short-range connectivity in the corresponding lobe.

### Decreased gyrification

We used a validated technique with exquisite resolution to measure local cortical gyrification across the hemispheres (Schaer et al., 2008), and observed four clusters of reduced GI in patients with ASD compared to controls, three of them located in the frontal lobe. This is in contrast with previous studies using GI in children, adolescents or adults with ASD, which report either an increased GI (Hardan et al., 2004; Jou et al., 2010), or an absence of significant difference (Casanova et al., 2009; Kates et al., 2009; Meguid et al., 2010). Lower intellectual abilities in our patient group may explain part of the divergence with previous results, given that both studies that reported increased GI comprised participants with higher full-scale IQ scores (means: 105 ± 16 for Hardan et al. (2004), 110 ± 15 for Jou et al. (2010) and 113 ± 15 for Wallace et al. (2013)). However, we believe part of the difference to relate to the way GI was calculated. Indeed, the two studies that reported higher GI in the frontal lobe of subjects with ASD used manual delineation on one single frontal slice. Aside from the fact that manual tracing may be less reliable than automated delineation, measuring GI on 2-D sections does not take into account the inherent 3-D nature of the cortical surface. 2-D measurement also can be biased by slice orientation (Zilles et al., 1997) and the presence of buried sulci (Magnotta et al., 1999), and it does not allow for precise localization of gyral anomalies in sublobar regions. Other studies that partly addressed these concerns did not report any significant differences in GI in patients with ASD compared to controls (Casanova et al., 2009; Kates et al., 2009; Meguid et al., 2010). Casanova et al. used manual delineation in 40 randomly selected slices; Kates et al. applied an automated technique for measuring global and lobar GI based on 2D sections; and Meguid et al. measured global GI using three-dimensional cortical reconstructions. The technique that we use in the present study to measure *l*GI is automated, unbiased by slice orientation, and allows the quantification of gyrification differences at thousands of points over the reconstructed cortical surface. As a result, by using *l*GI, we may have been able to detect gyrification differences in the frontal lobe of patients with ASD that had previously gone undetected. Studies using cortical reconstructions in autism corroborate this idea. They have detected focal changes to sulcal shape with alterations to the sylvian fissure and inferior frontal sulcus (Levitt et al., 2003), left frontal operculum (Nordahl et al., 2007), and right intraparietal sulcus (Shokouhi et al., 2012). It is however worth noting that a recent study using the same technique as in the present study observed increased gyrification in different regions of the brain of 39 male adolescents with ASD as compared to 41 controls, namely in bilateral occipital areas as well as in the left superior precuneus (Wallace et al., 2013). This discrepancy in the location and direction of gyrification changes using the same technique suggest either that different developmental mechanisms take place in different regions of the brain of affected patients, or that that demographic characteristics (such as differences in age, gender, cognitive level, or symptom intensity) may have influenced the results. Indeed, it may be the case that the high clinical heterogeneity observed in patients affected with autism may be associated with different neurodevelopmental pathways.

Decreased gyrification, as observed in the present study, is highly suggestive of reduced cortical expansion during early brain development, a process that might differentially affect specific cortical regions. Neuropathological reports have pointed to abnormal cortical development in ASD, including a higher incidence of cortical dysgenesis, heterotopias and migration abnormalities (Avino and Hutsler, 2010; Wegiel et al., 2010). Further detailed examination revealed that one cell type affected by migration deficits in young children with ASD is von Economo neurons (Santos et al., 2011), which are spindle-shaped neurons thought to play a role in emotional function (Butti et al., 2013) that are located in the frontoinsular and cingulate cortices. The location of the von Economo neurons coincides with the location of cluster 3 in the present study (see Figure 2). This anterior fronto-insular region is attracting increased attention in autism because of its key role in the “salience network” (Menon and Uddin, 2010). The anterior insula may have a critical role in processing information relevant to social functioning (Uddin and Menon, 2009) as a sort of “hub” that mediates interactions between cerebral networks that are processing information related to an external or internal stimulus. Functional neuroimaging studies tend to confirm the hypothesis of hypoactivation of the right anterior insula in autism, as pointed out by a meta-analysis based on 24 functional neuroimaging studies examining social processes for a total of 276 patients with ASD and 291 controls (Di Martino et al., 2009).

Two other clusters, in the right inferior frontal gyrus (cluster 2) and in a region extending from the right inferior part of the precentral gyrus to the inferior parietal region (cluster 1), are also located in regions that have received attention in ASD. Indeed, these regions are striking in their correspondence to the location of the fronto-parietal mirror neuron system, implicated in action imitation (Rizzolatti and Craighero, 2004). Decreased gyrification in the inferior fronto-parietal region thus supports altered development of the mirror system in ASD during *in utero* life or the first months after birth, pointing to a potential mechanism for early-disrupted abilities to imitate action of others.

The final cluster of reduced gyrification (cluster 4) is centered in the occipital lobe, encompassing the cuneus and the pericalcarine sulcus, and further extending to the precuneus. Volumetric reductions have been consistently reported in the precuneus in structural neuroimaging studies of ASD (Cauda et al., 2011). The precuneus is a key part of the default mode network (DMN), which is thought to be concerned with self-referential and introspective activity, including the ability to understand others’ intentions (Fair et al., 2008). Resting state paradigms have received increased recent interest in autism given the crucial role of the DMN in some aspects of social cognition. It is currently unclear to what extent the fronto-parietal mirror system (where we observed decreased *l*GI in clusters 1 and 2) interacts with other regions of the social brain, including regions of the DMN. In an attempt to integrate these different views of the social brain, (Uddin et al., 2007) postulated that the cortical midline structures of the DMN and the fronto-parietal mirror-neuron system may represent two interwoven parts of self-related processing and social cognition: the mirror neurons encode physical aspects of social understanding (motor simulation and imitation of behaviors) and the midline DMN structures are associated with sophisticated processing of social interactions. Accordingly, reduced gyrification in the mirror-neuron system may impair physical aspects of the self-other relationship, consequently altering the developmental cascade of the DMN responsible for more sophisticated social skills, such as empathy and theory of mind. The fact that cluster 4 extends into the precuneus may also point to an early defect, on top of which altered cortical maturation subsequently occurs. Indeed, the currently observed inverse relationship between gyrification and the level of autistic symptoms in the domains of reciprocal social interactions and communication points to the idea that early cortical development may determine the subsequent development of these more sophisticated social skills encoded in the precuneus.

### Absence of cortical thickness or volume difference

We found reduced gyrification in the absence of differences in cortical thickness or volume. This absence contrasts with numerous studies that have reported altered cortical volume or thickness differences in ASD. Most studies have reported increased cortical volume or thickness in children with ASD (Hardan et al., 2006; Mak-Fan et al., 2012), whereas studies in adults have yielded more diverse results. Some studies in adults with ASD have reported mostly decreased cortical thickness (Hadjikhani et al., 2006; Jiao et al., 2010; Wallace et al., 2010), some have shown a co-occurrence of thickening together with thinning (Ecker et al., 2010, 2013), and at least one has shown mostly thickening (Dziobek et al., 2010). Volumetric studies have more consistently reported increased volume in children and reduced volume in adults, supporting the hypothesis of early brain overgrowth followed by neurodegenerative changes (Courchesne, 2004). Indeed, using a cross-sectional design with patients aged 1 to 50, the largest study published to date provides evidence for an aberrant trajectory of cortical volume changes with age, with a pattern of early overgrowth during the first years of life, followed by decreased volume around 7 or 8 years old (Courchesne et al., 2011). Longitudinal studies confirm this pattern of abnormal cortical development in toddlers with ASD (Schumann et al., 2010) and of higher rates of cortical loss with age (Hardan et al., 2009). It should be noted, however, that studies recording abnormal trajectories of cortical features require large sample sizes, a broad age range at inclusion, and preferably, a longitudinal design. By contrast, our small study sample does not have the power to detect subtle cortical thickness differences that may be further diluted by complex maturational changes.

### Convergent evidence for altered long-range connectivity, most prominent in the frontal region

As the largest white matter bundle of the brain, the corpus callosum represents the most essential component of connectivity, and more specifically long-range connectivity. Several fMRI and EEG studies have reported decreased long-range connectivity (reviewed in Belmonte et al., 2004). Patients with ASD were also shown to perform poorly on tests of inter-hemispheric transfer for auditory, visual and motor tasks (Nyden et al., 2004). More generally, decreased abilities in associative (Nikolaenko, (2001a) and metaphoric thinking (Nikolaenko, 2001b) were thought to depend on decreased inter-hemispheric information transfer. Here, we observed decreased volume of the most anterior part of the corpus callosum, reduced inter-hemispheric frontal connections, and decreased FA in the anterior corpus callosum, providing strong multimodal evidence for altered inter-hemispheric frontal connections in ASD. Our volumetric finding is consistent with previous studies reporting reduced area of the entire corpus callosum, with greater magnitude of reduction in its anterior region (see the meta-analysis by Frazier and Hardan, 2009). Reduced FA in the corpus callosum is also consistent with many previous findings reported by others (Alexander et al., 2007; Noriuchi et al., 2010; Shukla et al., 2010). But, to the best of our knowledge, only one study reported a reduction in the number of inter-hemispheric frontal fibers using tractographic reconstructions in patients with ASD (Thomas et al., 2011). Thomas et al. observed decreased numbers of streamlines specific to the body in high-functioning adults with ASD, which further correlated with ADI scores in the domain of restricted, repetitive and stereotyped behaviors. The fact that we replicate this correlation (though we focus on a more anterior, but overlapping, region) provides strong support for a role for the corpus callosum in repetitive behaviors, across ages and across IQ.

### Reduced FA

In addition to multimodal evidence for altered inter-hemispheric connectivity, we also observed seven clusters of decreased FA in the right hemisphere of patients with ASD compared to controls. The direction of our results was consistent with most previously published studies, which show decreased FA, although a few studies do report increased FA (reviewed in Travers et al., 2012). Surprisingly, in our small sample of children and adolescents with ASD, we found reduced FA only in the right hemisphere and did not detect changes to FA in the left hemisphere. Exclusively right-sided alterations to gyrification in the same sample of participants provide initial support for a relationship between white matter connectivity and cortical folding. However, it was not possible to detect whether the observed FA differences were related to differences in the degree of myelinisation or to differences in the orientation or number of white matter bundles, using voxel-wise measurements of FA. The spatial correspondence of altered gyrification and white matter microstructure in the same hemisphere led us to further examine the relationship between cortical folding and connectivity using more sophisticated tractographic measurements.

### Correlation between gyrification and connectivity

We did not observe a relationship between long-range connectivity and gyrification, as may have been expected from Van Essen’s hypothesis that mechanical tension exerted on long connections shapes cortical folds (Van Essen, 1997). However, three out of the four clusters with decreased gyrification were mostly located in the frontal region, i.e., the region where an important decrease in inter-hemispheric connectivity was observed. The co-occurrence of decreased long-range connections in regions of altered gyrification points to a possible relationship between these two anatomical variables, but the mechanisms governing their association is likely to be more complex than what a linear regression can capture.

We did, however, observe significant positive correlations between *l*GI and short-range connectivity in patients with ASD, but not in controls. This positive correlation means that higher *l*GI was observed in patients with higher intra-lobar (short-range) connectivity. According to Van Essen’s theory, it may also be that short-range connections affect the creation of cortical folds during early brain development by reducing the distance between strongly interconnected regions from the two banks of one gyrus, thereby permitting compact wiring of the brain. Accordingly, the gyrification alterations observed in the present study may be a compensatory way of coping with altered connectivity in patients with ASD.

### Limitations and conclusion

The main limitation of our study is its small sample size, restricting our ability to identify age-related maturational changes or subtle brain-behavior relationships. We realize that, in a heterogeneous disorder such as ASD, such small sample size may lead to observation of findings that may not be representative of the variability observed across the spectrum. However, despite the small sample size, we demonstrate the feasibility of multimodal studies in autism, bridging the gap between reports of altered cortical morphometry and findings of abnormal connectivity patterns. These preliminary results provide initial support for the idea that a higher degree of short-range connectivity alters the shape of the brain in patients with ASD during early neural development, and are an encouraging starting point for exploring this issue in larger samples of children, adolescents or adults with autism.

## Conflict of interest statement

The authors declare that the research was conducted in the absence of any commercial or financial relationships that could be construed as a potential conflict of interest.

## Footnotes

^1^http://surfer.nmr.mgh.harvard.edu

^2^http://fsl.fmrib.ox.ac.uk/fsl/fslwiki/

^3^http://www.connectomics.org/connectomemapper/

^4^http://trackvis.org/dtk/

## References

[B1] Alexander A. L. Lee J. E. Lazar M. Boudos R. Dubray M. B. Oakes T. R. (2007). Diffusion tensor imaging of the corpus callosum in Autism. Neuroimage 34, 61–73 10.1016/j.neuroimage.2006.08.032 17023185

[B2] Assaf M. Jagannathan K. Calhoun V. D. Miller L. Stevens M. C. Sahl R. (2010). Abnormal functional connectivity of default mode sub-networks in autism spectrum disorder patients. Neuroimage 53, 247–256 10.1016/j.neuroimage.2010.05.067 20621638 PMC3058935

[B3] Avino T. A. Hutsler J. J. (2010). Abnormal cell patterning at the cortical gray-white matter boundary in autism spectrum disorders. Brain Res. 1360, 138–146 10.1016/j.brainres.2010.08.091 20816758

[B4] Barttfeld P. Wicker B. Cukier S. Navarta S. Lew S. Sigman M. (2011). A big-world network in ASD: dynamical connectivity analysis reflects a deficit in long-range connections and an excess of short-range connections. Neuropsychologia 49, 254–263 10.1016/j.neuropsychologia.2010.11.024 21110988

[B5] Belmonte M. K. Allen G. Beckel-Mitchener A. Boulanger L. M. Carper R. A. Webb S. J. (2004). Autism and abnormal development of brain connectivity. J. Neurosci. 24, 9228–9231 10.1523/jneurosci.3340-04.2004 15496656 PMC6730085

[B6] Berument S. K. Rutter M. Lord C. Pickles A. Bailey A. (1999). Autism screening questionnaire: diagnostic validity. Br. J. Psychiatry 175, 444–451 10.1192/bjp.175.5.444 10789276

[B7] Butti C. Santos M. Uppal N. Hof P. R. (2013). Von Economo neurons: clinical and evolutionary perspectives. Cortex 49, 312–326 10.1016/j.cortex.2011.10.004 22130090

[B8] Casanova M. F. El-Baz A. Mott M. Mannheim G. Hassan H. Fahmi R. (2009). Reduced gyral window and corpus callosum size in autism: possible macroscopic correlates of a minicolumnopathy. J. Autism Dev. Disord. 39, 751–764 10.1007/s10803-008-0681-4 19148739 PMC2911778

[B9] Cauda F. Geda E. Sacco K. D’agata F. Duca S. Geminiani G. (2011). Grey matter abnormality in autism spectrum disorder: an activation likelihood estimation meta-analysis study. J. Neurol. Neurosurg. Psychiatry 82, 1304–1313 10.1136/jnnp.2010.239111 21693631

[B88] Centers for Disease Control and Prevention (2008). “Prevalence of autism spectrum disorders - autism and developmental disabilities monitoring network, 14 sites, United States 2008” by Jon Baio. Centers for Disease Control and Prevention Morbidity and Mortality Weekly Report, Vol. 61, 1–19 22456193

[B10] Courchesne E. (2004). Brain development in autism: early overgrowth followed by premature arrest of growth. Ment. Retard. Dev. Disabil. Res. Rev. 10, 106–111 10.1002/mrdd.20020 15362165

[B11] Courchesne E. Campbell K. Solso S. (2011). Brain growth across the life span in autism: age-specific changes in anatomical pathology. Brain Res. 1380, 138–145 10.1016/j.brainres.2010.09.101 20920490 PMC4500507

[B12] Courchesne E. Karns C. M. Davis H. R. Ziccardi R. Carper R. A. Tigue Z. D. (2001). Unusual brain growth patterns in early life in patients with autistic disorder: an MRI study. Neurology 57, 245–254 10.1212/wnl.57.2.245 11468308

[B13] Daducci A. Gerhard S. Griffa A. Lemkaddem A. Cammoun L. Gigandet X. (2012). The connectome mapper: an open-source processing pipeline to map connectomes with MRI. PLoS One 7:e48121 10.1371/journal.pone.0048121 23272041 PMC3525592

[B14] Dale A. M. Fischl B. Sereno M. I. (1999). Cortical surface-based analysis. I. Segmentation and surface reconstruction. Neuroimage 9, 179–194 10.1006/nimg.1998.0395 9931268

[B15] Desikan R. S. Segonne F. Fischl B. Quinn B. T. Dickerson B. C. Blacker D. (2006). An automated labeling system for subdividing the human cerebral cortex on MRI scans into gyral based regions of interest. Neuroimage 31, 968–980 10.1016/j.neuroimage.2006.01.021 16530430

[B16] Di Martino A. Ross K. Uddin L. Q. Sklar A. B. Castellanos F. X. Milham M. P. (2009). Functional brain correlates of social and nonsocial processes in autism spectrum disorders: an activation likelihood estimation meta-analysis. Biol. Psychiatry 65, 63–74 10.1016/j.biopsych.2008.09.022 18996505 PMC2993772

[B17] Dziobek I. Bahnemann M. Convit A. Heekeren H. R. (2010). The role of the fusiform-amygdala system in the pathophysiology of autism. Arch. Gen. Psychiatry 67, 397–405 10.1001/archgenpsychiatry.2010.31 20368515

[B18] Ecker C. Ginestet C. Feng Y. Johnston P. Lombardo M. V. Lai M. C. (2013). Brain surface anatomy in adults with autism: the relationship between surface area, cortical thickness, and autistic symptoms. JAMA Psychiatry 70, 59–70 10.1001/jamapsychiatry.2013.265 23404046

[B19] Ecker C. Marquand A. Mourao-Miranda J. Johnston P. Daly E. M. Brammer M. J. (2010). Describing the brain in autism in five dimensions–magnetic resonance imaging-assisted diagnosis of autism spectrum disorder using a multiparameter classification approach. J. Neurosci. 30, 10612–10623 10.1523/jneurosci.5413-09.2010 20702694 PMC6634684

[B20] Elsabbagh M. Divan G. Koh Y. J. Kim Y. S. Kauchali S. Marcin C. (2012). Global prevalence of autism and other pervasive developmental disorders. Autism Res. 5, 160–179 10.1002/aur.239 22495912 PMC3763210

[B21] Fair D. A. Cohen A. L. Dosenbach N. U. Church J. A. Miezin F. M. Barch D. M. (2008). The maturing architecture of the brain’s default network. Proc. Natl. Acad. Sci. U S A 105, 4028–4032 10.1073/pnas.0800376105 18322013 PMC2268790

[B22] Fischl B. (2012). FreeSurfer. Neuroimage 62, 774–781 22248573 10.1016/j.neuroimage.2012.01.021 PMC3685476

[B23] Fischl B. Dale A. M. (2000). Measuring the thickness of the human cerebral cortex from magnetic resonance images. Proc. Natl. Acad. Sci. U S A 97, 11050–11055 10.1073/pnas.200033797 10984517 PMC27146

[B24] Fischl B. Sereno M. I. Dale A. M. (1999). Cortical surface-based analysis. II: Inflation, flattening, and a surface-based coordinate system. Neuroimage 9, 195–207 10.1006/nimg.1998.0396 9931269

[B25] Frazier T. W. Hardan A. Y. (2009). A meta-analysis of the corpus callosum in autism. Biol. Psychiatry 66, 935–941 10.1016/j.biopsych.2009.07.022 19748080 PMC2783565

[B26] Hadjikhani N. Joseph R. M. Snyder J. Tager-Flusberg H. (2006). Anatomical differences in the mirror neuron system and social cognition network in autism. Cereb. Cortex 16, 1276–1282 10.1093/cercor/bhj069 16306324

[B27] Han X. Jovicich J. Salat D. Van Der Kouwe A. Quinn B. Czanner S. (2006). Reliability of MRI-derived measurements of human cerebral cortical thickness: the effects of field strength, scanner upgrade and manufacturer. Neuroimage 32, 180–194 10.1016/j.neuroimage.2006.02.051 16651008

[B28] Hardan A. Y. Jou R. J. Keshavan M. S. Varma R. Minshew N. J. (2004). Increased frontal cortical folding in autism: a preliminary MRI study. Psychiatry Res. 131, 263–268 10.1016/j.pscychresns.2004.06.001 15465295

[B29] Hardan A. Y. Libove R. A. Keshavan M. S. Melhem N. M. Minshew N. J. (2009). A preliminary longitudinal magnetic resonance imaging study of brain volume and cortical thickness in autism. Biol. Psychiatry 66, 320–326 10.1016/j.biopsych.2009.04.024 19520362 PMC2905654

[B30] Hardan A. Y. Muddasani S. Vemulapalli M. Keshavan M. S. Minshew N. J. (2006). An MRI study of increased cortical thickness in autism. Am. J. Psychiatry 163, 1290–1292 10.1176/appi.ajp.163.7.1290 16816240 PMC1509104

[B31] Haukvik U. K. Schaer M. Nesvag R. Mcneil T. Hartberg C. B. Jonsson E. G. (2011). Cortical folding in Broca’s area relates to obstetric complications in schizophrenia patients and healthy controls. Psychol. Med. 42, 1329–1337 10.1017/S0033291711002315 22029970

[B32] Jiao Y. Chen R. Ke X. Chu K. Lu Z. Herskovits E. H. (2010). Predictive models of autism spectrum disorder based on brain regional cortical thickness. Neuroimage 50, 589–599 10.1016/j.neuroimage.2009.12.047 20026220 PMC2823830

[B33] Jou R. J. Minshew N. J. Keshavan M. S. Hardan A. Y. (2010). Cortical gyrification in autistic and Asperger disorders: a preliminary magnetic resonance imaging study. J. Child Neurol. 25, 1462–1467 10.1177/0883073810368311 20413799 PMC3115701

[B34] Juranek J. Salman M. S. (2010). Anomalous development of brain structure and function in spina bifida myelomeningocele. Dev. Disabil. Res. Rev. 16, 23–30 10.1002/ddrr.88 20419768 PMC2917986

[B35] Kates W. R. Ikuta I. Burnette C. P. (2009). Gyrification patterns in monozygotic twin pairs varying in discordance for autism. Autism Res. 2, 267–278 10.1002/aur.98 19890876

[B36] Kennedy D. P. Courchesne E. (2008). The intrinsic functional organization of the brain is altered in autism. Neuroimage 39, 1877–1885 10.1016/j.neuroimage.2007.10.052 18083565

[B37] Le Couteur A. Lord C. Rutter M. L. (2007). Entretien pour le Diagnostic de l’Autisme - version révisée, adaptation française de Bernadette Rogé et collaborateurs. Editions Hogrefe France.

[B38] Levitt J. G. Blanton R. E. Smalley S. Thompson P. M. Guthrie D. Mccracken J. T. (2003). Cortical sulcal maps in autism. Cereb. Cortex 13, 728–735 10.1093/cercor/13.7.728 12816888

[B39] Lord C. Rutter M. L. Dilavore P. C. Risi S. (2009). ADOS: Echelle d’observation pour le diagnostic de l’autisme, adaptation française par Bernadette Rogé et collaborateurs. Editions Hogrefe France.

[B40] Magnotta V. A. Andreasen N. C. Schultz S. K. Harris G. Cizadlo T. Heckel D. (1999). Quantitative in vivo measurement of gyrification in the human brain: changes associated with aging. Cereb. Cortex 9, 151–160 10.1093/cercor/9.2.151 10220227

[B41] Mak-Fan K. M. Taylor M. J. Roberts W. Lerch J. P. (2012). Measures of cortical grey matter structure and development in children with autism spectrum disorder. J. Autism Dev. Disord. 42, 419–427 10.1007/s10803-011-1261-6 21556969

[B42] Meguid N. Fahim C. Yoon U. Nashaat N. H. Ibrahim A. S. Mancini-Marie A. (2010). Brain morphology in autism and fragile X syndrome correlates with social IQ: first report from the Canadian-Swiss-Egyptian neurodevelopmental study. J. Child Neurol. 25, 599–608 10.1177/0883073809341670 20110214

[B43] Menon V. Uddin L. Q. (2010). Saliency, switching, attention and control: a network model of insula function. Brain Struct. Funct. 214, 655–667 10.1007/s00429-010-0262-0 20512370 PMC2899886

[B44] Minshew N. J. Williams D. L. (2007). The new neurobiology of autism: cortex, connectivity, and neuronal organization. Arch. Neurol. 64, 945–950 10.1001/archneur.64.10.1464 17620483 PMC2597785

[B45] Mori S. Crain B. J. Chacko V. P. Van Zijl P. C. (1999). Three-dimensional tracking of axonal projections in the brain by magnetic resonance imaging. Ann. Neurol. 45, 265–269 10.1002/1531-8249(199902)45:2<265::aid-ana21>3.0.co;2-3 9989633

[B46] Nichols T. E. Holmes A. P. (2002). Nonparametric permutation tests for functional neuroimaging: a primer with examples. Hum. Brain Mapp. 15, 1–25 10.1002/hbm.1058 11747097 PMC6871862

[B47] Nikolaenko N. N. (2001a). Associative process as an indicator of interhemispheric interaction in healthy children and children with autism. Dokl. Biol. Sci. 380, 430–432 10.1023/A:1012354801160 12918395

[B48] Nikolaenko N. N. (2001b). Metaphorical thinking in healthy and autistic children as an index of interhemispheric interaction. Dokl. Biol. Sci. 379, 325–327 10.1023/A:1011639927334 12918365

[B49] Nordahl C. W. Dierker D. Mostafavi I. Schumann C. M. Rivera S. M. Amaral D. G. (2007). Cortical folding abnormalities in autism revealed by surface-based morphometry. J. Neurosci. 27, 11725–11735 10.1523/jneurosci.0777-07.2007 17959814 PMC6673212

[B50] Noriuchi M. Kikuchi Y. Yoshiura T. Kira R. Shigeto H. Hara T. (2010). Altered white matter fractional anisotropy and social impairment in children with autism spectrum disorder. Brain Res. 1362, 141–149 10.1016/j.brainres.2010.09.051 20858472

[B51] Nyden A. Carlsson M. Carlsson A. Gillberg C. (2004). Interhemispheric transfer in high-functioning children and adolescents with autism spectrum disorders: a controlled pilot study. Dev. Med. Child Neurol. 46, 448–454 10.1017/s001216220400074x 15230457

[B52] Palaniyappan L. Liddle P. F. (2012). Differential effects of surface area, gyrification and cortical thickness on voxel based morphometric deficits in schizophrenia. Neuroimage 60, 693–699 22227049 10.1016/j.neuroimage.2011.12.058

[B53] Palaniyappan L. Mallikarjun P. Joseph V. White T. P. Liddle P. F. (2011). Folding of the prefrontal cortex in schizophrenia: regional differences in gyrification. Biol. Psychiatry 69, 974–979 10.1016/j.biopsych.2010.12.012 21257157

[B54] Rakic P. (1988). Specification of cerebral cortical areas. Science 241, 170–176 10.1126/science.3291116 3291116

[B55] Regis J. Mangin J. F. Ochiai T. Frouin V. Riviere D. Cachia A. (2005). “Sulcal root” generic model: a hypothesis to overcome the variability of the human cortex folding patterns. Neurol. Med. Chir. (Tokyo) 45, 1–17 10.2176/nmc.45.1 15699615

[B56] Richman D. P. Stewart R. M. Hutchinson J. W. Caviness Jr. V. S. (1975). Mechanical model of brain convolutional development. Science 189, 18–21 10.1126/science.1135626 1135626

[B57] Rizzolatti G. Craighero L. (2004). The mirror-neuron system. Annu. Rev. Neurosci. 27, 169–192 10.1146/annurev.neuro.27.070203.144230 15217330

[B58] Ronan L. Scanlon C. Murphy K. Maguire S. Delanty N. Doherty C. P. (2011). Cortical curvature analysis in MRI-negative temporal lobe epilepsy: a surrogate marker for malformations of cortical development. Epilepsia 52, 28–34 10.1111/j.1528-1167.2010.02895.x 21198558

[B59] Rosas H. D. Lee S. Y. Bender A. C. Zaleta A. K. Vangel M. Yu P. (2010). Altered white matter microstructure in the corpus callosum in Huntington’s disease: implications for cortical “disconnection”. Neuroimage 49, 2995–3004 10.1016/j.neuroimage.2009.10.015 19850138 PMC3725957

[B60] Rosas H. D. Liu A. K. Hersch S. Glessner M. Ferrante R. J. Salat D. H. (2002). Regional and progressive thinning of the cortical ribbon in Huntington’s disease. Neurology 58, 695–701 10.1212/wnl.58.5.695 11889230

[B61] Salat D. H. Greve D. N. Pacheco J. L. Quinn B. T. Helmer K. G. Buckner R. L. (2009). Regional white matter volume differences in nondemented aging and Alzheimer’s disease. Neuroimage 44, 1247–1258 10.1016/j.neuroimage.2008.10.030 19027860 PMC2810540

[B62] Santos M. Uppal N. Butti C. Wicinski B. Schmeidler J. Giannakopoulos P. (2011). Von Economo neurons in autism: a stereologic study of the frontoinsular cortex in children. Brain Res. 1380, 206–217 10.1016/j.brainres.2010.08.067 20801106

[B63] Schaer M. Cuadra M. B. Tamarit L. Lazeyras F. Eliez S. Thiran J. P. (2008). A surface-based approach to quantify local cortical gyrification. IEEE Trans. Med. Imaging 27, 161–170 10.1109/tmi.2007.903576 18334438

[B64] Schaer M. Glaser B. Cuadra M. B. Debbane M. Thiran J. P. Eliez S. (2009). Congenital heart disease affects local gyrification in 22q11.2 deletion syndrome. Dev. Med. Child Neurol. 51, 746–753 10.1111/j.1469-8749.2009.03281.x 19416334

[B65] Schumann C. M. Bloss C. S. Barnes C. C. Wideman G. M. Carper R. A. Akshoomoff N. (2010). Longitudinal magnetic resonance imaging study of cortical development through early childhood in autism. J. Neurosci. 30, 4419–4427 10.1523/jneurosci.5714-09.2010 20335478 PMC2859218

[B66] Shokouhi M. Williams J. H. Waiter G. D. Condon B. (2012). Changes in the sulcal size associated with autism spectrum disorder revealed by sulcal morphometry. Autism Res. 5, 245–252 10.1002/aur.1232 22674695

[B67] Shukla D. K. Keehn B. Lincoln A. J. Muller R. A. (2010). White matter compromise of callosal and subcortical fiber tracts in children with autism spectrum disorder: a diffusion tensor imaging study. J. Am. Acad. Child Adolesc. Psychiatry 49, 1269–1278, e1261–e1262 10.1016/j.jaac.2010.08.018 21093776 PMC3346956

[B68] Smith S. M. Jenkinson M. Johansen-Berg H. Rueckert D. Nichols T. E. Mackay C. E. (2006). Tract-based spatial statistics: voxelwise analysis of multi-subject diffusion data. Neuroimage 31, 1487–1505 10.1016/j.neuroimage.2006.02.024 16624579

[B69] Smith S. M. Johansen-Berg H. Jenkinson M. Rueckert D. Nichols T. E. Miller K. L. (2007). Acquisition and voxelwise analysis of multi-subject diffusion data with tract-based spatial statistics. Nat. Protoc. 2, 499–503 10.1038/nprot.2007.45 17406613

[B70] Srivastava S. Buonocore M. H. Simon T. J. (2012). Atypical developmental trajectory of functionally significant cortical areas in children with chromosome 22q11.2 deletion syndrome. Hum. Brain Mapp. 33, 213–223 10.1002/hbm.21206 21416559 PMC3212617

[B71] Thesen T. Quinn B. T. Carlson C. Devinsky O. Dubois J. Mcdonald C. R. (2011). Detection of epileptogenic cortical malformations with surface-based MRI morphometry. PLoS One 6:e16430 10.1371/journal.pone.0016430 21326599 PMC3033882

[B72] Thomas C. Humphreys K. Jung K. J. Minshew N. Behrmann M. (2011). The anatomy of the callosal and visual-association pathways in high-functioning autism: a DTI tractography study. Cortex 47, 863–873 10.1016/j.cortex.2010.07.006 20832784 PMC3020270

[B73] Travers B. G. Adluru N. Ennis C. Tromp Do P. M. Destiche D. Doran S. (2012). Diffusion tensor imaging in autism spectrum disorder: a review. Autism Res. 5, 289–313 10.1002/aur.1243 22786754 PMC3474893

[B74] Uddin L. Q. Iacoboni M. Lange C. Keenan J. P. (2007). The self and social cognition: the role of cortical midline structures and mirror neurons. Trends Cogn. Sci. 11, 153–157 10.1016/j.tics.2007.01.001 17300981

[B75] Uddin L. Q. Menon V. (2009). The anterior insula in autism: under-connected and under-examined. Neurosci. Biobehav. Rev. 33, 1198–1203 10.1016/j.neubiorev.2009.06.002 19538989 PMC2743776

[B76] Van Essen D. C. (1997). A tension-based theory of morphogenesis and compact wiring in the central nervous system. Nature 385, 313–318 10.1038/385313a0 9002514

[B77] von dem Hagen E. A. Stoyanova R. S. Baron-Cohen S. Calder A. J. (2013). Reduced functional connectivity within and between ‘social’ resting state networks in autism spectrum conditions. Soc. Cogn. Affect Neurosci. 8, 694–701 22563003 10.1093/scan/nss053 PMC3739917

[B78] Wallace G. L. Dankner N. Kenworthy L. Giedd J. N. Martin A. (2010). Age-related temporal and parietal cortical thinning in autism spectrum disorders. Brain 133, 3745–3754 10.1093/brain/awq279 20926367 PMC2995883

[B79] Wallace G. L. Robustelli B. Dankner N. Kenworthy L. Giedd J. N. Martin A. (2013). Increased gyrification, but comparable surface area in adolescents autism spectrum disorders. Brain 136, 1956–1967 10.1093/brain/awt106 23715094 PMC3673467

[B80] Wechsler D. (1991). Wechsler Intelligence Scale for Children - Third edition. Manual. San Antonio, TX: The Psychological Corporation

[B81] Wegiel J. Kuchna I. Nowicki K. Imaki H. Marchi E. Ma S. Y. (2010). The neuropathology of autism: defects of neurogenesis and neuronal migration, and dysplastic changes. Acta Neuropathol. 119, 755–770 10.1007/s00401-010-0655-4 20198484 PMC2869041

[B82] Welker W. (1990). “Why does cerebral cortex fissure and fold? A review of determinants of gyri and sulci,” in Cerebral Cortex, eds Jones E. G. Peters A. (New York: Plenum), 3–136

[B83] Weng S. J. Wiggins J. L. Peltier S. J. Carrasco M. Risi S. Lord C. (2010). Alterations of resting state functional connectivity in the default network in adolescents with autism spectrum disorders. Brain Res. 1313, 202–214 10.1016/j.brainres.2009.11.057 20004180 PMC2818723

[B84] Zhang Y. Yu C. Zhou Y. Li K. Li C. Jiang T. (2009). Decreased gyrification in major depressive disorder. Neuroreport 20, 378–380 10.1097/wnr.0b013e3283249b34 19218876

[B85] Zhang Y. Zhou Y. Yu C. Lin L. Li C. Jiang T. (2010). Reduced cortical folding in mental retardation. AJNR Am. J. Neuroradiol. 31, 1063–1067 10.3174/ajnr.a1984 20075096 PMC7963948

[B86] Zikopoulos B. Barbas H. (2010). Changes in prefrontal axons may disrupt the network in autism. J. Neurosci. 30, 14595–14609 10.1523/jneurosci.2257-10.2010 21048117 PMC3073590

[B87] Zilles K. Schleicher A. Langemann C. Amunts K. Morosan P. Palomero-Gallagher N. (1997). Quantitative analysis of sulci in the human cerebral cortex: development, regional heterogeneity, gender difference, asymmetry, intersubject variability and cortical architecture. Hum. Brain Mapp. 5, 218–221 10.1002/(SICI)1097-0193(1997)5:4<218::AID-HBM2>3.0.CO;2-6 20408218

